# Practical Applicability of the ISARIC-4C Score on Severity and Mortality due to SARS-CoV-2 Infection in Patients with Type 2 Diabetes

**DOI:** 10.3390/medicina58070848

**Published:** 2022-06-25

**Authors:** Oana Albai, Mirela Frandes, Alexandra Sima, Bogdan Timar, Adrian Vlad, Romulus Timar

**Affiliations:** 1 Department of Internal Medicine-Diabetes, Nutrition, Metabolic Diseases and Systemic Rheumatology, “Victor Babes” University of Medicine and Pharmacy, 300041 Timisoara, Romania; albai.oana@umft.ro (O.A.); sima.alexandra@umft.ro (A.S.); timar.bogdan@umft.ro (B.T.); vlad.adrian@umft.ro (A.V.); timar.romulus@umft.ro (R.T.); 2 Department of Diabetes and Metabolic Diseases, “Pius Brinzeu” Emergency Hospital, 300182 Timisoara, Romania; 3 Centre for Molecular Research in Nephrology and Vascular Disease/MOL-NEPHRO-VASC, “Victor Babes” University of Medicine and Pharmacy, 300041 Timisoara, Romania; 4 Department of Functional Sciences—Medical Informatics and Biostatistics, “Victor Babes” University of Medicine and Pharmacy, 300041 Timisoara, Romania

**Keywords:** SARS-CoV-2 infection, ISARIC-4C score, diabetes mellitus, risk, severity, mortality

## Abstract

*Background and objectives*. There is a bidirectional relationship between SARS-CoV-2 infection and diabetes mellitus (DM), as people with DM are more vulnerable, and SARS-CoV-2 infections worsen the prognosis in these patients. The main purpose of the study was to evaluate the application validity of the ISARIC-4C score in patients confirmed with SARS-CoV-2 infection. *Materials and Methods*. The study included 159 patients previously known to have type 2 DM and confirmed positive for SARS-CoV-2 infection. We analyzed the concordance between the clinical evaluation of the patients and the ISARIC-4C score. *Results*. The mortality rate in hospitalized patients was 25.15%. The mortality risk was higher for ISARIC-4C values >14 than in the opposite group (63.93% vs. 31.24%; *p* < 0.001). The area under the curve (AUC) of the mortality score was 0.875 (95% CI: 0.820–0.930; *p* < 0.001), correctly classifying 77.36% of the cohort. A cut-off value of >14 had a sensitivity of 87.80% (95% CI: 87.66–87.93), specificity 73.72% (95% CI: 73.48–73.96), positive predictive value 53.73% (95% CI: 53.41–54.04), and negative predictive value 94.56% (95% CI: 94.5–94.62). The Cox regression model showed that the length of hospitalization (LH) was significantly influenced by body mass index, lung impairment, and aspartate aminotransferase, increasing the hazards, while lower HbA1c and lower SatO_2_ significantly decreased the hazards. *Conclusions*. ISARIC-4C score estimates the likelihood of clinical deterioration and the mortality risk in patients hospitalized with type 2 DM and positive for SARS-CoV-2, being useful in assessing the prognosis from the onset, as well as in developing therapeutic strategies.

## 1. Introduction

Severe acute respiratory syndrome coronavirus 2 (SARS-CoV-2) infection emerged in December 2019 in the city of Wuhan, from where it spread around the world, creating a pandemic. There are currently over 450 million cases worldwide and over 6 million deaths [1]. Patients with diabetes mellitus (DM) are vulnerable, both physically and psychologically, and are much more prone to infections [2]. Unsatisfactory glycemic control, chronic complications, other associated comorbidities, and cardiovascular risk factors (CVRs) have been shown to increase the risk of SARS-CoV-2 infection 2–3-fold in patients with DM [3,4]. DM is a condition involving the immune and cardiovascular systems, and it is associated with various disorders of platelet function and clotting, making the body vulnerable to SARS-CoV-2. Moreover, the presence of comorbidities (e.g., high blood pressure, obesity, ischemic cardiomyopathy) and specific complications of DM (cardiovascular events, such as myocardial infarction, stroke, renal failure, lower limb arteriopathy) negatively influence the course of the infectious disease [5,6,7,8,9,10].

SARS-CoV-2 is an RNA virus that has 82% homology to the human SARS-CoV virus that causes severe acute respiratory syndrome (SARS) [11]. It is a single-stranded RNA virus consisting of four structural proteins, namely, spike glycoprotein (S), an envelope protein (E), membrane protein (M) and nucleocapsid (N). SARS-CoV-2 glycoprotein S allows the virus to enter host cells using the angiotensin-converting enzyme 2-ACE2 receptor. ACE2 receptors are found in large amounts in the respiratory tract and lung parenchyma, as well as in key metabolic organs (e.g., muscle, liver, endocrine pancreas, fat tissue), which play an important role in glucose homeostasis. The known spike protein of the coronavirus could attack and destroy β-pancreatic cells, like DM [12,13,14]. Thus, the SARS-CoV-2 infection causes altered insulin secretion by impaired ß-pancreatic cell function or inflammation-induced insulin resistance, with the triggering of pleiotropic effects that affect glucose metabolism, exacerbating a pre-existing DM or triggering *de novo* DM [15]. 

Infection with SARS-CoV-2 is accompanied by the release of excessive amounts of proinflammatory cytokines and chemokines: interferon, TNF-alpha, interleukins 1beta, 6, 12, 18, 33, and others, which trigger a violent immune response against the lungs (causing acute respiratory distress syndrome (ARDS)) and other organs (leading to multiple organ failure), which rapidly progress to death. Moreover, increased levels of IL6 and lactate dehydrogenase (LDH) are direct indicators of disease severity [16,17]. Moreover, patients with coronavirus disease (COVID-19) show increased values of other inflammatory markers (D-Dimers, ferritin) that contribute to an increased risk of micro and macrovascular complications. Glycemic deterioration is a complication of COVID-19, with SARS-CoV-2 infection requiring significant dose increases in insulin-treated patients [18]. These changes appear to be associated with pro-inflammatory cytokine levels [19]. Although diabetic ketoacidosis (DKA) is a more common acute metabolic complication in type 1 DM, case analysis has shown that approximately 77% of SARS-CoV-2-associated DKA infections occurred in patients with type 2 DM [20].

The ISARIC-4C score (4C Mortality Score and 4C Deterioration models) represents risk stratification tools that predict in-hospital mortality or in-hospital clinical deterioration. It is designed to be easy to use, requiring only parameters that are commonly available at hospital admission. The ISARIC-4C score took into consideration the following variables: nosocomial (defined as the onset of symptoms or the first positive SARS-CoV-2), sex, number of comorbidities (chronic heart disease; chronic respiratory diseases-excluding asthma; chronic kidney disease-estimated glomerular filtration rate ≤30; mild to severe liver disease; dementia; chronic neurological disorders; connective tissue disease; diabetes mellitus-diet, tablet or insulin-controlled; HIV-AIDS; malignancy; obesity), radiographic chest infiltrates, receiving oxygen (when oxygen saturation measured), Glasgow coma scale (<15 or >15), age (years), respiratory rate (breaths/min), admission oxygen saturation (%), urea (mmol/L), CRP (mg/L), lymphocytes (×10⁹/L) [21].

The main purpose of this study was to evaluate the validity of the ISARIC-4C score application in patients with type 2 DM, confirmed with SARS-CoV-2 infection and hospitalized at the emergency department. We analyzed the concordance between the clinical evaluation and the ISARC-4C score, following other risk factors that may influence the prognosis of these patients.

## 2. Material and Methods

### 2.1. Study Design and Patients

**Figure 1:**
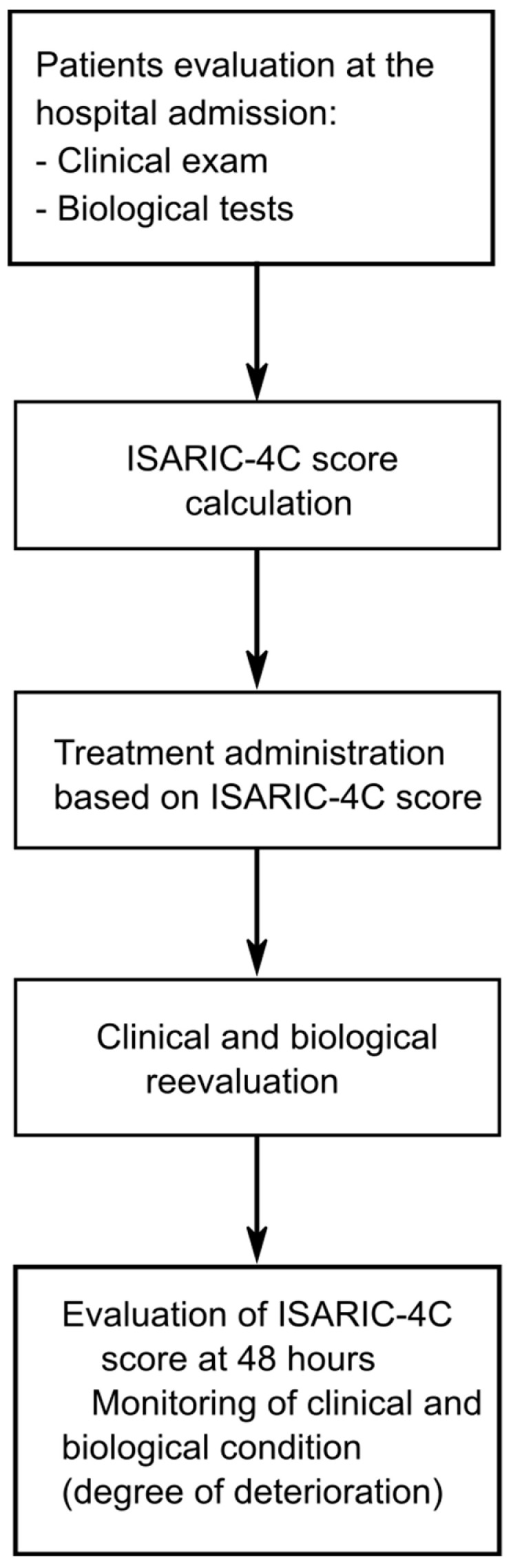
Diagram representing the process of the ISARIC-4C score application.

The study included 159 patients admitted to “Pius Brînzeu” County Hospital in Timisoara through the emergency department, from 1 December 2020 to 1 March 2021. Patients were previously known for having type 2 DM. After coming to the emergency room, patients were confirmed positive for SARS-CoV-2 infection and admitted to the special ward for COVID-19 patients with DM. Patients who died in the first 14 days after admission were classified as non-survivors. The exclusion criteria were under age 18-year-old patients, patients with severe psychiatric disorders and patients who presented major acute cardiovascular events. The diagram representing the process of the ISARIC-4C score application is presented in Figure 1. 

This study was conducted in accordance with the Declaration of Helsinki, and the protocol of our study was approved by the Ethics Committee of the Emergency County Hospital Timisoara (Approval number: 164/16 November 2020). All patients included in the study provided written informed consent for the acquisition, analysis, and publication of the anonymous data collected during their hospital admission.

### 2.2. Clinical, Anthropometric, and Laboratory Data

What was collected: demographics, family history and past medical history, associated malignancies, clinical and paraclinical investigations, and treatment administered. Evaluation of glycemic control was performed by inspecting fasting, postprandial glycaemia and HbA1c. The lipid panel (e.g., CT, TG, HDLc, LDLc) was also assessed. The presence of DM-related complications was also investigated: microangiopathic (e.g., diabetic retinopathy, diabetic renal disease), macroangiopathic (e.g., ischemic heart disease, diabetic arteriopathy, neurovascular damage), as well as neuropathic ones. Patients were interviewed and the presence of previously known associated pulmonary pathology was noted (e.g., chronic obstructive pulmonary disease, bronchial asthma, bronchopulmonary cancer). For assessing the degree of lung damage, all patients underwent a pulmonary CT scan, both at admission and at discharge.

Clinical parameters (e.g., blood pressure, heart rate, respiratory rate, oxygen saturation—SatO_2_, temperature, diuresis), as well as paraclinical parameters (e.g., C-reactive protein—CRP, fibrinogen, complete blood count, D-Dimer, liver tests—ASAT, ALAT, serum ferritin, urea, creatinine) were dynamically monitored.

A thorough history was taken, specifying the exact reasons for admission (e.g., cough, myalgia, headache, odynophagia, muscle weakness, fever, chills, nausea, vomiting, diarrhea, etc.).

### 2.3. Confirmation of SARS-CoV-2 Infection

For the detection of SARS-CoV-2, we used the reference test known as SARS-CoV-2 viral RNA, which uses the Real-Time PCR method (RT-PCR). Polymerase chain reaction (PCR) detects the virus through techniques of amplification of viral genetic material. Samples from the upper respiratory tract (nasal and pharyngeal exudate) were collected, preferably together with blood and other samples from the lower respiratory tract (sputum or bronchial alveolar lavage—in case of severe disease). From these samples, viral identification is performed by the RT-PCR method. The sample is considered positive if two coronavirus-specific gene fragments are detected, one of which is specific to SARS-CoV-2.

The clinical forms of SARS-CoV-2 infection are: Mild form: patient with any signs and symptoms (fever, cough, sore throat, malaise, headache, muscle aches) without difficulty breathing, dyspnea or abnormal chest images (without pneumonia). Moderate form: patient with fever and signs of non-severe pneumonia, without the need for additional oxygen. Severe form: patient with signs of severe pneumonia. Severe adult pneumonia: suspected, probable, or confirmed case of COVID 19, plus one of the following: • severe dyspnea (FR ≥ 30/min); • hypoxemia (SpO2 ≤ 94% at rest); • PaO2/FiO2 ≤ 300 mmHg; • rapid negative evolution of the lung imaging pattern in the last 24–48 h by ≥50%; • progressive decrease in the number of peripheral lymphocytes and rapid increase in lactate. Critical form: patients with COVID-19 and one or more of the following: ARDS; shock; any organ failure that requires care in the intensive care unit; other conditions with major danger to the patient’s life.

### 2.4. ISARIC-4C Score

The severity was assessed by computing the ISARIC-4C score, which aids the clinician in making therapeutic decisions. The ISARIC score allows objective assessment of severity and risk of death based on clinical and paraclinical characteristics. It is a risk stratification score of mortality or deterioration of clinical status. Herein, clinical deterioration means the need for ventilatory support, critical care in an intensive care unit, or death. 

The computation of the ISARIC-4C score includes the following parameters: age, gender, number of present comorbidities, lung impairment, O_2_ requirement, Glasgow score, respiratory rate/min, O_2_ saturation, serum urea, C-reactive protein, lymphocyte count (score range 0–21 points) [21]. 

### 2.5. Statistical Analysis

The data were described by computing the mean ± standard deviation for continuous variables and the frequency (percentage) for nominal variables. A comparison of survivor and non-survivor groups was made considering the type of variables. Continuous variables were compared using the t-test or Wilcoxon rank-sum test, while nominal variables were compared using the chi-square or Fisher’s exact test. The normality of continuous variable was tested using the Kolmogorov–Smirnov’s test. 

We analyzed the mortality rate by plotting the Receiver Operator Characteristic (ROC) curve using the ISARIC-4C score as the classification variable. We presented the ROC curve together with the significance level *p*. Applying Youden’s index criterion, we determined the optimal cut-off value we used in the mortality analysis. 

Kaplan–Meier survival curves were presented for the days of hospitalization of each risk group based on the ISARIC-4C score. A Cox proportional hazards regression model was derived to assess the effect of risk factors on the length of hospitalization. We entered the variables which were previously identified as being significantly different in the group of survivors compared with non-survivors into the model. We compared the days of hospitalization of each risk group considering the hazard ratios (HR) computed by the Cox regression model. Harrell’s C-index, the concordance index, was used as a goodness of fit measure for models.

The statistical analysis was performed with MedCalc^®^ Statistical Software version 20.106 (MedCalc Software Ltd., Ostend, Belgium). The significance threshold was considered *p*-value < 0.05.

## 3. Results

### 3.1. Characteristics of Patients

In the study period (1st December 2020 to 1st March 2021), 159 admissions of COVID-19 confirmed cases were recorded through the emergency room. On the 159 patients included in the study, 85 were male (53.45%), and 74 were female (46.55%). Patients were previously diagnosed with type 2 DM. The mean age was 68.05 ± 10.06 years, and the average duration of DM was 9.98 ± 4.71 years. The general characteristics of the patients are presented in Table 1. 

A percentage of 25.15% were hospital deaths. The mortality score was 13.66 ± 3.19, while the death risk was 45% ± 19.15. The deterioration score was 633.68 ± 159.83, while the deterioration risk was 67.37% ± 19.09. 

The age of survivors was significantly lower than non-survivors (65.20 ± 8.75 vs. 70.90 ± 9.30; *p* = 0.010). Moreover, significant differences were noticed for SatO_2_, lung damage, respiratory rate, PCR, D-dimers, ASAT, ALAT, urea. 

Hypertension has been identified in almost all patients: 157 patients (98.74%). Of these, 48.42% presented Hypertension grade II and 50.31% grade III. Cardiac pathology (pectoris angina, atrial fibrillation, heart failure) was present in 152 patients (95.59%). Moreover, 32 patients (20.12%) had a history of lung disease (chronic obstructive pulmonary disease, asthma) and 6 patients (3.77%) had a known neoplasm. Table 2 shows the associated comorbidities. 

### 3.2. Accuracy of the ISARIC-4C Score

**Figure 2:**
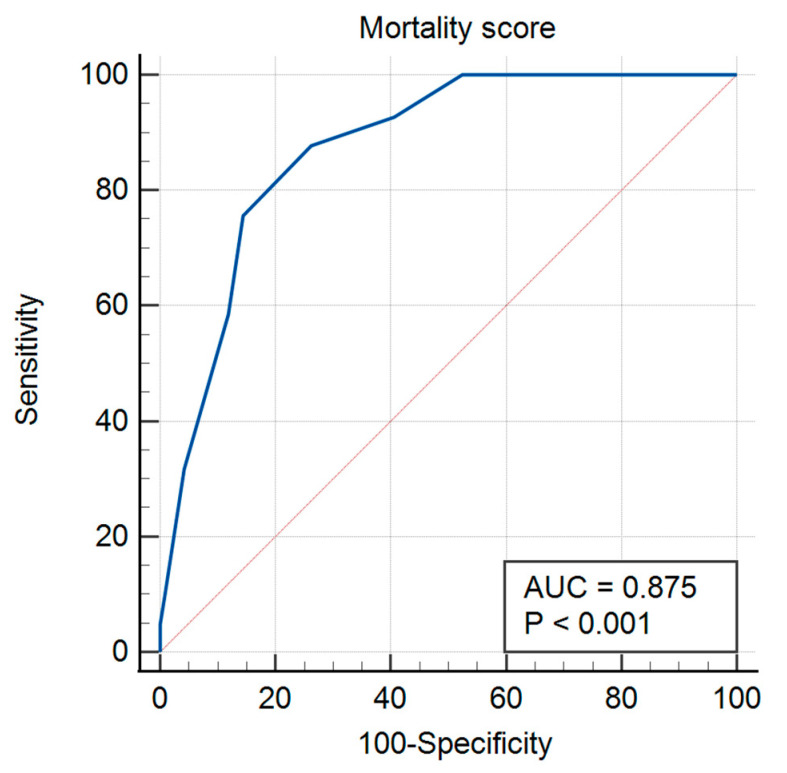
Receiver-operating characteristics (ROC) analysis of the mortality score. Abbreviations: AUC, Area under the curve.

The ROC curve of the mortality score had AUC of 0.875 (95% CI: 0.820–0.930; *p* < 0.001) (Figure 2).

Applying the Youden’s index, we identified the optimal cut-off value of the ISARIC score as > 14. The sensitivity associated with the optimal cut-off score >14 was 87.80% (95% CI: 87.66–87.93), the specificity was 73.72% (95% CI: 73.48–73.96), while the predictive values (PV) were positive PV of 53.73% (95% CI: 53.41–54.04), and negative PV of 94.56% (95% CI: 94.5–94.62). 

When grouping the cohort according to the ISARIC cut-off score, we observed a higher mortality score for values >14 than in the group with scores equal or less than 14 (16.49 vs. 11.61; *p* < 0.001). Moreover, the mortality risk was higher for ISARIC values > 14 than in the group with scores equal or less than 14 (62.67% vs. 32.01%; *p* < 0.001). When considering the deterioration, we observed a higher deterioration score for ISARIC values > 14 than in the opposite group (744.42 vs. 532.22; *p* < 0.001). The deterioration risk was also higher for ISARIC values > 14 than in the opposite group (82.08 vs. 54.83; *p* < 0.001). A percent of 87.8% of patients with ISARIC scores higher than 14 were non-survivors vs. 12.2% of patients with ISARIC scores less than 14 were non-survivors (*p* < 0.001). Most of the patients with ISARIC scores higher than 14 were at ICM/OTI (85.7%), while 14.3% of patients with ISARIC scores less than 14 were at ICM/OTI (14.3%) (*p* < 0.001). 

### 3.3. Hospitalization Rate

**Figure 3:**
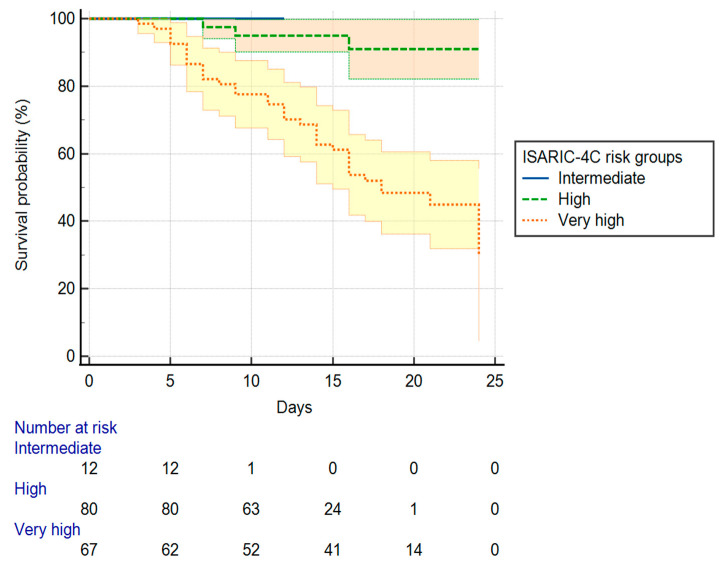
Kaplan–Meier curve of hospitalization days in surviving patients grouped by the ISARIC-4C risk.

We compared the days of hospitalization among survivors for the intermediate (scores: 4–8), high (scores: 9–14), and very high-risk (scores: ≥15) groups by the ISARIC-4C mortality score (Figure 3).

The mean of hospitalization days for the intermediate-risk group was 12 days (95% CI: 12.00–12.00), while for the high-risk and very high-risk groups it was 22.88 days (95% CI: 21.91–23.85), and 17.16 days (15.39–18.93). We observed a significant difference between the hospitalization days of the risk groups (Log-rank test χ^2^(2) = 25.593, *p* < 0.001). The high-risk group had a significantly lower HR = 0.151 (95% CI: 0.081–0.282) for discharge than the intermediate-risk group, while in the very high-risk group the HR was 6.612 (95% CI: 3.543–12.338).

The overall model fit of the Cox regression showed a significant relationship between the days of hospitalization and the covariates in the model (Chi-square test χ^2^(12) = 136.075, *p* < 0.001). The Harrell’s C-index was 0.878 (95% CI: 0.842–0.914) indicating that the model is good at predicting which patients will require a longer length of hospitalization. The multivariate Cox regression analysis showed that age did not influence the days of hospitalization, HR = 0.981 (95% CI: 0.921–1.046). We observed that BMI had significantly influenced the days of hospitalization increasing the hazard, HR = 1.183 (95% CI: 1.015–1.381). On the contrary, a lower mean of HbA1c significantly influenced the days of hospitalization lowering the hazard, HR = 0.641 (95% CI: 0.492–0.835) (Table 3). 

The lung impairment significantly influenced the days of hospitalization increasing the hazard, HR = 1.075 (95% CI: 1.029–1.124). In addition, SatO2 significantly influenced the days of hospitalization lowering the hazard, HR = 0.871 (95% CI: 0.814–0.932). Moreover, ALAT significantly influenced the days of hospitalization increasing the hazard, HR = 1.011 (95% CI: 1.002–1.021). 

## 4. Discussion

Patients with DM, especially those with poor glycemic control and chronic complications are at higher risk of becoming infected with the SARS-CoV-2 virus and developing severe forms of the disease. Since the beginning of the pandemic, DM has been reported as one of the increased risk factors responsible for the rapid progression and poor prognosis of COVID-19 [22]. In general, patients with DM have a higher susceptibility to infectious diseases such as tuberculosis, pneumonia, or influenza. The mechanisms involved are not completely known, but it seems that an important role is played by chronic hyperglycemia, as the hyperglycemic environment is known to increase the virulence of some pathogens. Phagocytosis and chemotactic processes have been shown to be impaired, and the T-cell, neutrophil responses and interleukin secretion are reduced [22,23,24,25].

We report an ISARIC-4C score > 14 as an indicator of higher mortality risk in patients hospitalized with type 2 DM and positive for SARS-CoV-2. The mortality rate in our group was 25.15%, the mean age being statistically significantly higher in patients who died, compared to survivors: 70.90 ± 9.30 years versus 65.20 ± 8.75 years (*p* = 0.010). In our group, patients were previously known with type 2 DM, with a mean duration of disease of 9.98 ± 4.71 years. We also found a rather high value for mean blood sugar at admission, 228.97 ± 106.56 mg/dL.

Yang et al. compared data from over 400 patients and found that serum levels of fasting blood glucose were higher in patients with SARS than in patients with pneumonia without SARS. Moreover, 21.5% of patients who had a poor outcome of SARS infection had a history of DM, a significantly higher percentage than those who recovered (3.9%) [16]. A study conducted in Wuhan showed that of 26 deaths caused by SARS-CoV-2, 42.3% had DM [26]. Another study showed that COVID-19-related death was associated with being male (hazard ratio (HR) 1.59 (95% confidence interval 1.53–1.65)), greater age and deprivation (both with a strong gradient), diabetes, severe asthma, and various other medical conditions. Compared with people of white ethnicity, black and south Asian people were at higher risk, even after adjustment of other factors (HR 1.48 (1.29–1.69) and 1.45 (1.32–1.58), respectively) [27]. Another cohort study that looked at 201 patients in Wuhan with confirmed COVID-19 pneumonia showed that 10.9% of patients had DM. They also noted that among the patients who developed ARDS (41.8%), DM was the 2nd most common comorbidity (19.0% in ARDS patients versus 5.1% in non-ARDS patients) [28].

Kulcsar et al. studied the severity of MERS-CoV infection in a group of mice in which DM was induced by a high-fat diet [29]. Results showed a more severe and prolonged acute phase of infection in mice with DM with altered recruitment of lung CD4+ T cells and altered secretion of cytokines particularly increased IL17. A similar immune response is observed in patients with COVID-19, with reduced CD4+ T cell counts and increased Th17 cell counts. It is known that the production of IL2 decreases with age, which favors the differentiation of CD4+ T cells into Th17 [30]. Moreover, this inflammatory profile may explain the severity of SARS-CoV-2 infection in patients with DM [31].

A multicenter retrospective study conducted in China has shown that fasting blood sugar ≥ 126 mg/dL at admission was an independent predictor of increased mortality in patients with COVID-19 without DM [32]. Severe acute respiratory syndrome from SARS-CoV-2 infection results in tissue hypoxia with interstitial lung injury and acute respiratory distress syndrome [33]. Patients with DM and COVID-19 have a higher risk of clinical deterioration and faster progression to cardiorespiratory failure precisely because of increased oxidative stress, cytokine production and endothelial dysfunction. All these characteristic changes in patients with DM are accompanied by higher thromboembolic risk and faster deterioration of vital organs [34,35,36,37,38]. 

Among all patients under observation, the percentage of those presenting respiratory manifestations (dyspnea, irritative cough, headache, myalgia, fever) was significantly higher than those presenting digestive symptomatology at onset (nausea, vomiting, diarrhea). Most of the patients had hypertension or another cardiac pathology. Using the ISARIC-4C score to assess the risk of clinical deterioration, as well as the risk of mortality, we identified a cut-off value of 14: the sensitivity associated with this value was 87.80% and the specificity 73.72%. 

A limitation of our study is that we included the evaluation of the ISARIC-4C score performance, without testing the efficiency of an individualized treatment based on this score. Moreover, patients without DM or patients newly diagnosed with DM were not included. The strengths of our study include the chosen population, i.e., patients with DM, who present complex and diverse pathology with a high risk of multiple complications. Therefore, applying a prognostic score is of great importance.

## 5. Conclusions

Patients with DM need close and careful monitoring to ensure optimal glycemic control, thus preventing major metabolic imbalances, such as diabetic ketoacidosis, or other complications, both acute and chronic. This appears to be the solution to prevent clinical deterioration and poor prognosis in the case of patients with COVID-19. In the studied group, the application of the ISARIC-4C score was useful for assessing the prognosis from the moment of hospitalization and the mortality risk in patients hospitalized with type 2 DM and positive for SARS-CoV-2, being useful in developing therapeutic strategies.

## Figures and Tables

**Table 1:** General characterization of patients: anthropometric parameters, lipidic and glycemic profiles at admission.

Variable	All Cohort (n = 159)	Survivors (n = 117)	Non-Survivors (n = 42)	*p*-Value
Age (years)	68.05 ± 10.06	65.20 ± 8.75	70.90 ± 11.30	0.010
Gender (Males)	85 (53.45%)	63 (53.4%)	22 (53.7%)	0.976
BMI (kg/m^2^)	31.24 ± 4.31	30.34 ± 3.76	32.14 ± 4.63	0.0134
Duration of DM (years)	9.98 ± 4.71	9.76 ± 4.45	10.20 ± 4.98	0.5947
Mean HbA1c (%)	9.02 ± 1.06	8.84 ± 0.78	9.20 ± 1.36	0.0394
Mean blood sugar at admission (mg/dL)	228.97 ± 106.56	211.19 ± 104.98	246.75 ± 114.43	0.0675
Serum cholesterol (mg/dL)	206.01 ± 39.08	200.88 ± 37.23	211.14 ± 40.19	0.1351
Serum triglycerides (mg/dL)	245.43 ± 117.68	214.52 ± 114.34	276.34 ± 119.76	0.0034
HDLc (mg/dL)	36.62 ± 12.64	38.17 ± 13.88	35.06 ± 54.12	0.5654
LDLc (mg/dL)	121.36 ± 32.81	119.80 ± 35.17	122.92 ± 37.56	0.6284
Fever	151 (94.96%)	112 (94.9%)	39 (95.1%)	0.838
Dyspnea	157 (98.74%)	118 (100%)	39 (95.1%)	0.016
Myalgia	142 (89.3%)	103 (87.3%)	39 (95.1%)	0.132
Diarrhea	10 (6.28%)	4 (3.4%)	6 (14.6%)	0.019
Nausea/vomiting	9 (5.66%)	6 (5.1%)	3 (7.3%)	0.422
SatO_2_ (%)	84.93 ± 8.26	93.53 ± 8.43	76.33 ± 5.72	<0.001
Lung impairment (%)	48.83 ± 17.73	43.55 ± 11.31	54.11 ± 18.86	<0.001
Respiratory rate-RR	30.18 ± 4.93	28.07 ± 3.74	32.29 ± 5.01	<0.001
PCR	157.41 ± 81.56	100.09 ± 52.96	214.73 ± 94.17	<0.001
D-Dimer	1257.79 ± 142.06	925.97 ± 115.98	1589.61 ± 193.92	<0.001
ASAT	53.91 ± 24.71	44.73 ± 20.22	63.09 ± 30.98	<0.001
ALAT	57.46 ± 51.90	44.21 ± 17.03	70.71 ± 96.23	<0.001
Lymphocytes	983.90 ± 538.36	896.34 ± 443.51	1071.46 ± 739.02	0.112
Low platelets	294,981 ± 96,013.54	300,762.71 ± 95,017.82	278,341.46 ± 98,097.05	0.226
Urea	52.40 ± 31.67	47.11± 23.06	57.69 ± 45.68	<0.001
Mortality score	13.66 ± 3.19	12.63 ± 2.94	14.69 ± 1.74	<0.001
Risk of death (%)	45.18 ± 19.15	38.72 ± 16.84	51.64 ± 11.98	<0.001
Deterioration score	633.68 ± 159.83	610.42 ± 108.65	656.94 ± 162.76	<0.001
Risk of deterioration (%)	67.37 ± 19.09	59.56 ± 15.58	75.18 ± 5.70	<0.001
ICM/OTI	42 (26.4%)	2 (1.7%)	40 (97.6%)	<0.001

Abbreviations: BMI, body mass index; HDLc, HDL cholesterol; LDLc, LDL cholesterol; SatO2, oxygen saturation; PCR, C-reactive protein; ALAT, alanine aminotransferase; ASAT, aspartate aminotransferase; ICM/OTI, intensive care measures/oro-tracheal intubation.

**Table 2:** Associated comorbidities.

Comorbidity	Type	Value
Hypertension	Grade II	77 (48.42%)
	Grade III	80 (50.31%)
Neoplasm	Breast cancer	1 (0.62%)
	Melanoma	1 (0.62%)
	Laryngeal cancer	1 (0.62%)
	Bronchopulmonary neoplasm	1 (0.62%)
	Prostate cancer	1 (0.62%)
	Gastric neoplasm	1 (0.62%)
Heart Disease	Chronic coronary heart disease: pectoris angina, atrial fibrillation, heart failure	152 (95.59%)
Lung disease	Chronic obstructive pulmonary disease	20 (12.57%)
	Asthma	9 (5.66%)

Note: Hypertension Grade II: 160–179/100–109 mmHg, Hypertension Grade III: ≥180/≥110 mmHg. Heart disease: angina pectoris, arrhythmias, heart failure. Lung disease: chronic obstructive pulmonary disease, asthma.

**Table 3:** Factors influencing the length of hospitalization.

Covariate	b	SEE	Wald	HR	95% CI	*p*-Value
Age (years)	−0.018	0.032	0.312	0.981	0.921–1.046	0.576
BMI (kg/m^2^)	0.168	0.078	4.632	1.183	1.015–1.381	0.031
HbA1c (%)	−0.443	0.135	10.821	0.641	0.492–0.835	0.001
Triglycerides (mg/dL)	0.001	0.002	0.136	1.001	0.995–1.006	0.712
Lung impairment (%)	0.072	0.022	10.397	1.075	1.029–1.124	0.001
SatO_2_ (%)	−0.138	0.034	16.081	0.871	0.814–0.932	<0.001
PCR	0.004	0.002	2.803	1.004	0.999–1.011	0.094
ASAT	−0.006	0.011	0.412	0.993	0.973–1.014	0.521
ALAT	0.011	0.004	5.882	1.011	1.002–1.021	0.015
D-Dimer	0.001	0.001	0.391	1.001	0.999–1.004	0.532
Urea	−0.008	0.005	2.681	0.991	0.981–1.002	0.101

Abbreviations: b, regression coefficient; SEE, standard error; Wald, Wald statistic (b/SE)^2^, HR, hazards ratio; 95% CI, 95% confidence interval for HR; BMI, body mass index; SatO_2_, oxygen saturation; PCR, C-reactive protein; ALAT, alanine aminotransferase; ASAT, aspartate aminotransferase.

## Data Availability

The data presented in this study are available on request from the corresponding author.
